# Crystal structure of 1-benzyl-4-formyl-1*H*-pyrrole-3-carb­oxamide

**DOI:** 10.1107/S2056989016000128

**Published:** 2016-01-09

**Authors:** Qi-Di Zhong, Sheng-Quan Hu, Hong Yan

**Affiliations:** a College of Life Science and Bio-Engineering, Beijing University of Technology, 100124 Chaoyang District, Beijing, People’s Republic of China

**Keywords:** crystal structure, pyrrole derivative, hydrogen bonding, C—H⋯π inter­actions

## Abstract

In the title compound, C_13_H_12_N_2_O_2_ (I), the mean planes of the pyrrole and benzyl rings are approximately perpendicular, forming a dihedral angle of 87.07 (4) °. There is an intra­molecular N—H⋯O hydrogen bond forming an S(7) ring motif. In the crystal, mol­ecules are linked *via* a pair of N—H⋯O hydrogen bonds forming inversion dimers. C—H⋯O hydrogen bonds link the dimers into chains along direction [10-1]. The chains are further linked by weak C—H⋯π inter­actions forming layers parallel to the *ac* plane.

## Abstract 2

In the title compound, the mean planes of the pyrrole and benzyl rings are almost normal to one another with a dihedral angle of 87.07 (4)°. In the crystal, mol­ecules are linked *via* a pair of N—H⋯O hydrogen bonds forming inversion dimers. C—H⋯O hydrogen bonds link the dimers into chains propagating along [10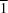].

## Chemical context   

Pyrrole and its derivatives are classes of heterocyclic compounds and that have attracted much attention because of their potential pharmacological and biological properties (Davis *et al.*, 2008 ▸; Meshram *et al.*, 2010 ▸; Moriguchi *et al.*, 2015 ▸). As a part of our work on the synthesis of new pyrrole derivatives with good biological activities, the title compound, (I), was synthesized and its crystal structure is reported on herein.
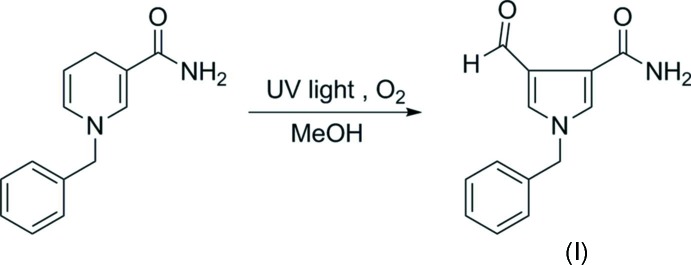


## Structural commentary   

**Figure 1:**
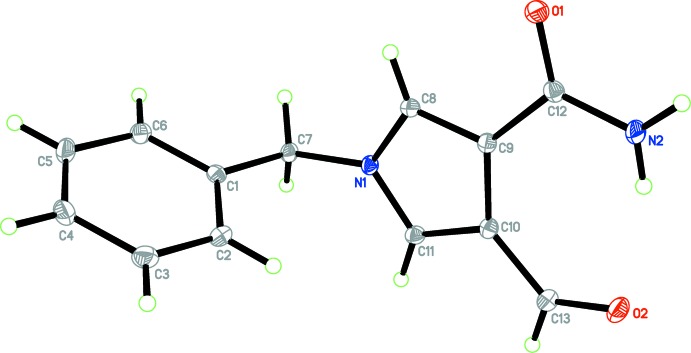
A view of the mol­ecular structure of the title compound (I), with atom labelling. Displacement ellipsoids are drawn at the 30% probability level.

The mol­ecular structure of the title compound (I), is shown in Fig. 1 ▸. In the amide group, the C—N bond is relatively short [C12—N2 = 1.3374 (16) Å], suggesting some degree of electronic delocalization in the mol­ecule. The dihedral angle between the pyrrole and phenyl rings is 87.07 (4)°, indicating that they are nearly perpendicular to each other. An intra­molecular hydrogen bond, N2—H2*B*⋯O2 (Table 1 ▸), encloses an *S*(7) ring motif.

## Supra­molecular features   

**Figure 2:**
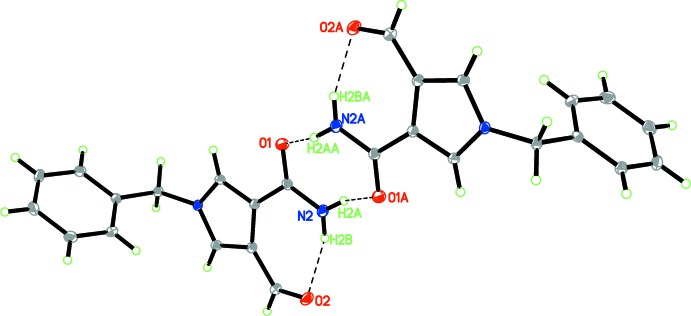
A view of the inversion dimer formed by pairs of N—H⋯O hydrogen bonds. Both the intra­molecular and inter­molecular hydrogen bonds are shown as dashed lines (see Table 1 ▸).

**Figure 3:**
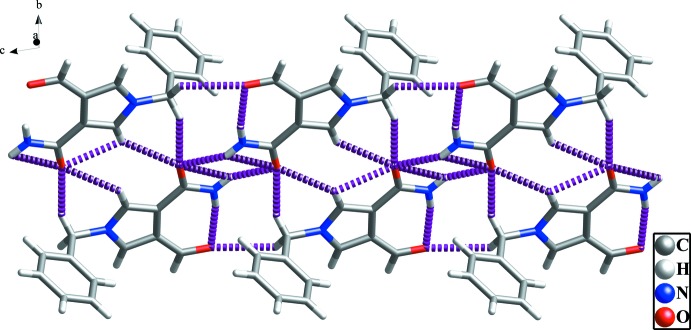
A view of the one-dimensional chain structure. The dashed lines indicate the N—H⋯O and C—H⋯O hydrogen bonds (see Table 1 ▸).

**Figure 4:**
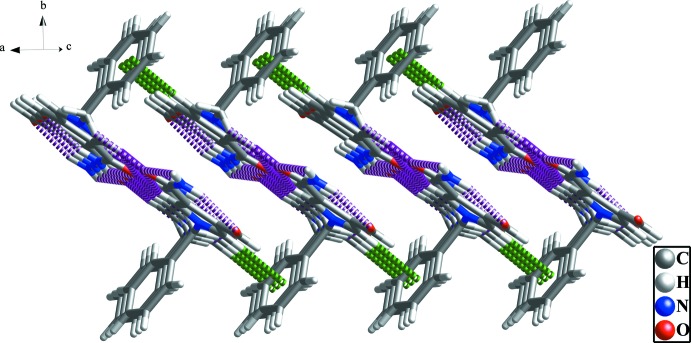
The view of the two-dimensional network structure. The C—H⋯π inter­actions and the hydrogen bonds are shown with green and purple dashed lines, respectively (see Table 1 ▸).

In the crystal of (I), N2—H2*A*⋯O1^i^ hydrogen bonds [symmetry code: (i) −*x* + 1, −*y* + 2, −*z* − 1], link pairs of mol­ecules, forming inversion dimers with an 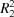
(8) ring motif (Table 1 ▸ and Fig. 2 ▸). The dimers are further linked by C7—H7*B*⋯O1^ii^, C8—H8⋯O1^ii^ and C7—H7*A*⋯O2^iii^ hydrogen bonds [symmetry codes: (ii) −*x* + 2, −*y* + 2, −*z*; (iii) *x* + 1, *y*, *z* + 1] into supra­molecular chains propagating along [10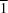]; see Table 1 ▸ and Fig. 3 ▸). Adjacent chains are linked by weak C11—H11⋯*Cg*1^iv^ contacts [*Cg*1 is the centroid of the C1—C6 benzyl ring; symmetry code: (iv) − 1 + *x*, *y*, *z*], forming layers parallel to the *ac* plane (Table 1 ▸ and Fig. 4 ▸).

## Database survey   

A search of the Cambridge Structural Database (Version 5.36 with three updates; Groom & Allen, 2014) for 1-benzyl-4-formyl-1*H*-pyrrole-3-carb­oxamide gave no hits. However, structures of substituted derivatives of 1-benzyl-1*H*-pyrrole were found, see for example Bonnett *et al.* (1985 ▸); Choi *et al.* (1998 ▸); Sha *et al.* (1990 ▸); Wang *et al.* (2011 ▸). In these structures, the pyrrole and benzyl rings are also nearly perpendicular to one another.

## Synthesis and crystallization   

1-Benzyl-1*H*-pyrrole-3-carb­oxamide (1 mmol, 214.3 mg) was dissolved in methanol (20 ml) and irradiated with UV light at room temperature under oxygen (see Scheme). The reaction progress was monitored by thin layer chromatography (TLC). After completion, the solvent was removed under reduced pressure, and the residue was purified by chromatography on silica gel, using a mixed solvent of petroleum ether and ethyl acetate (10:1 ratio, *v*/*v*), to give the pure product. Colourless single crystals, suitable for X-ray diffraction analysis, were obtained by slow evaporation of a methanol solution of the title compound at room temperature.

## Refinement   

Crystal data, data collection and structure refinement details are summarized in Table 2 ▸. All H atoms were placed in idealized positions (C—H = 0.93–0.97 Å, N—H = 0.86 Å) and refined as riding atoms, with *U*_iso_(H) = 1.2*U*_eq_(N,C).

## Supplementary Material

Crystal structure: contains datablock(s) global, I. DOI: 10.1107/S2056989016000128/su5268sup1.cif

Structure factors: contains datablock(s) I. DOI: 10.1107/S2056989016000128/su5268Isup2.hkl

CCDC reference: 1445256

Additional supporting information:  crystallographic information; 3D view; checkCIF report

## Figures and Tables

**Table 1:** Hydrogen-bond geometry (Å, °) *Cg*1 is the centroid of the benzyl ring C1–C6.

*D*—H⋯*A*	*D*—H	H⋯*A*	*D*⋯*A*	*D*—H⋯*A*
N2—H2*B*⋯O2	0.86	1.99	2.8184 (14)	160
N2—H2*A*⋯O1^i^	0.86	2.22	3.0063 (14)	151
C8—H8⋯O1^ii^	0.93	2.69	3.4252 (15)	136
C7—H7*B*⋯O1^ii^	0.97	2.48	3.3123 (15)	144
C7—H7*A*⋯O2^iii^	0.97	2.66	3.3268 (15)	126
C11—H11⋯*Cg*1^iv^	0.93	2.58	3.4962 (14)	167

Symmetry codes: (i) 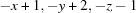; (ii) 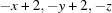; (iii) 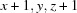; (iv) 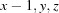.

**Table 2:** Experimental details

Crystal data	
Chemical formula	C_13_H_12_N_2_O_2_
*M*_r_	228.25
Crystal system, space group	Monoclinic, *P*2_1_/*c*
Temperature (K)	293
*a*, *b*, *c* (Å)	5.5296 (6), 23.083 (3), 9.3088 (9)
β (°)	112.940 (5)
*V* (Å^3^)	1094.2 (2)
*Z*	4
Radiation type	Mo *K*α
μ (mm^−1^)	0.10
Crystal size (mm)	0.25 × 0.20 × 0.18

Data collection	
Diffractometer	Bruker SMART CCD area detector
Absorption correction	Multi-scan (*SADABS*; Bruker, 2005 ▸)
*T*_min_, *T*_max_	0.977, 0.983
No. of measured, independent and observed [*I* > 2σ(*I*)] reflections	9372, 1938, 1823
*R*_int_	0.021
(sin θ/λ)_max_ (Å^−1^)	0.596

Refinement	
*R*[*F* ^2^ > 2σ(*F* ^2^)], *wR*(*F* ^2^), *S*	0.034, 0.123, 1.00
No. of reflections	1938
No. of parameters	154
H-atom treatment	H-atom parameters constrained
Δρ_max_, Δρ_min_ (e Å^−3^)	0.19, −0.26

Computer programs: *APEX2* and *SAINT* (Bruker, 2005 ▸) and *SHELXTL* (Sheldrick, 2008 ▸).

## supplementary crystallographic information

### Crystal data

**Table:**

C_13_H_12_N_2_O_2_	*F*(000) = 480
*M**_r_* = 228.25	*D*_x_ = 1.386 Mg m^−^^3^
Monoclinic, *P*2_1_/*c*	Mo *K*α radiation, λ = 0.71073 Å
Hall symbol: -P 2ybc	Cell parameters from 3196 reflections
*a* = 5.5296 (6) Å	θ = 3.5–27.5°
*b* = 23.083 (3) Å	µ = 0.10 mm^−^^1^
*c* = 9.3088 (9) Å	*T* = 293 K
β = 112.940 (5)°	Block, colorless
*V* = 1094.2 (2) Å^3^	0.25 × 0.20 × 0.18 mm
*Z* = 4	

### Data collection

**Table:**

Bruker SMART CCD area-detector diffractometer	1938 independent reflections
Radiation source: fine-focus sealed tube	1823 reflections with *I* > 2σ(*I*)
Graphite monochromator	*R*_int_ = 0.021
phi and ω scans	θ_max_ = 25.1°, θ_min_ = 3.5°
Absorption correction: multi-scan (*SADABS*; Bruker, 2005)	*h* = −6→6
*T*_min_ = 0.977, *T*_max_ = 0.983	*k* = −27→27
9372 measured reflections	*l* = −11→11

### Refinement

**Table:**

Refinement on *F*^2^	Primary atom site location: structure-invariant direct methods
Least-squares matrix: full	Secondary atom site location: difference Fourier map
*R*[*F*^2^ > 2σ(*F*^2^)] = 0.034	Hydrogen site location: inferred from neighbouring sites
*wR*(*F*^2^) = 0.123	H-atom parameters constrained
*S* = 1.00	*w* = 1/[σ^2^(*F*_o_^2^) + (0.1*P*)^2^ + 0.2118*P*] where *P* = (*F*_o_^2^ + 2*F*_c_^2^)/3
1938 reflections	(Δ/σ)_max_ = 0.001
154 parameters	Δρ_max_ = 0.19 e Å^−^^3^
0 restraints	Δρ_min_ = −0.26 e Å^−^^3^

### Special details

**Table:**

Geometry. All e.s.d.'s (except the e.s.d. in the dihedral angle between two l.s. planes) are estimated using the full covariance matrix. The cell e.s.d.'s are taken into account individually in the estimation of e.s.d.'s in distances, angles and torsion angles; correlations between e.s.d.'s in cell parameters are only used when they are defined by crystal symmetry. An approximate (isotropic) treatment of cell e.s.d.'s is used for estimating e.s.d.'s involving l.s. planes.
Refinement. Refinement of *F*^2^ against ALL reflections. The weighted *R*-factor *wR* and goodness of fit *S* are based on *F*^2^, conventional *R*-factors *R* are based on *F*, with *F* set to zero for negative *F*^2^. The threshold expression of *F*^2^ > σ(*F*^2^) is used only for calculating *R*-factors(gt) *etc*. and is not relevant to the choice of reflections for refinement. *R*-factors based on *F*^2^ are statistically about twice as large as those based on *F*, and *R*- factors based on ALL data will be even larger.

### Fractional atomic coordinates and isotropic or equivalent isotropic displacement parameters (Å^2^)

**Table:**

	*x*	*y*	*z*	*U*_iso_*/*U*_eq_	
C1	1.0335 (2)	0.84645 (5)	0.26085 (13)	0.0165 (3)	
C2	0.9577 (2)	0.80281 (5)	0.14829 (15)	0.0217 (3)	
H2	0.7904	0.8035	0.0696	0.026*	
C3	1.1293 (3)	0.75831 (6)	0.15255 (17)	0.0267 (3)	
H3	1.0774	0.7298	0.0760	0.032*	
C4	1.3792 (3)	0.75629 (6)	0.27120 (17)	0.0271 (3)	
H4	1.4942	0.7264	0.2746	0.033*	
C5	1.4545 (2)	0.79918 (6)	0.38396 (15)	0.0252 (3)	
H5	1.6204	0.7979	0.4640	0.030*	
C6	1.2845 (2)	0.84412 (5)	0.37859 (14)	0.0206 (3)	
H6	1.3385	0.8730	0.4543	0.025*	
C7	0.8542 (2)	0.89645 (5)	0.25832 (13)	0.0167 (3)	
H7A	0.7595	0.8869	0.3236	0.020*	
H7B	0.9601	0.9305	0.3022	0.020*	
C8	0.7226 (2)	0.94298 (5)	−0.00412 (13)	0.0158 (3)	
H8	0.8792	0.9627	0.0164	0.019*	
C9	0.5118 (2)	0.94220 (5)	−0.14451 (13)	0.0152 (3)	
C10	0.3150 (2)	0.90594 (5)	−0.12295 (13)	0.0162 (3)	
C11	0.4214 (2)	0.88767 (5)	0.03064 (13)	0.0171 (3)	
H11	0.3383	0.8638	0.0779	0.021*	
C12	0.5128 (2)	0.97561 (5)	−0.28041 (13)	0.0171 (3)	
C13	0.0602 (2)	0.88460 (5)	−0.22590 (14)	0.0199 (3)	
H13	−0.0288	0.8620	−0.1797	0.024*	
N1	0.66548 (19)	0.90998 (4)	0.10118 (11)	0.0152 (3)	
N2	0.2915 (2)	0.97471 (5)	−0.40897 (12)	0.0214 (3)	
H2A	0.2825	0.9936	−0.4906	0.026*	
H2B	0.1581	0.9553	−0.4100	0.026*	
O1	0.71039 (17)	1.00283 (4)	−0.27249 (10)	0.0237 (3)	
O2	−0.05271 (17)	0.89275 (4)	−0.36651 (10)	0.0255 (3)	

### Atomic displacement parameters (Å^2^)

**Table:**

	*U*^11^	*U*^22^	*U*^33^	*U*^12^	*U*^13^	*U*^23^
C1	0.0178 (6)	0.0173 (6)	0.0150 (6)	−0.0021 (5)	0.0072 (5)	0.0043 (4)
C2	0.0162 (6)	0.0221 (7)	0.0233 (7)	−0.0031 (5)	0.0041 (5)	−0.0016 (5)
C3	0.0249 (7)	0.0193 (7)	0.0347 (8)	−0.0027 (5)	0.0103 (6)	−0.0052 (5)
C4	0.0234 (7)	0.0199 (7)	0.0380 (8)	0.0052 (5)	0.0120 (6)	0.0061 (5)
C5	0.0181 (6)	0.0284 (7)	0.0244 (7)	0.0026 (5)	0.0033 (5)	0.0086 (5)
C6	0.0209 (6)	0.0228 (7)	0.0159 (6)	−0.0023 (5)	0.0046 (5)	0.0020 (5)
C7	0.0167 (6)	0.0198 (6)	0.0113 (6)	−0.0003 (5)	0.0029 (5)	0.0011 (4)
C8	0.0156 (6)	0.0153 (6)	0.0163 (6)	−0.0010 (4)	0.0062 (5)	0.0001 (4)
C9	0.0160 (6)	0.0142 (6)	0.0147 (6)	0.0013 (4)	0.0051 (5)	−0.0014 (4)
C10	0.0153 (6)	0.0175 (6)	0.0154 (6)	0.0012 (5)	0.0057 (5)	−0.0008 (4)
C11	0.0155 (6)	0.0183 (6)	0.0185 (6)	−0.0017 (5)	0.0076 (5)	0.0004 (5)
C12	0.0200 (6)	0.0144 (6)	0.0162 (6)	0.0009 (5)	0.0064 (5)	−0.0008 (4)
C13	0.0171 (6)	0.0221 (6)	0.0194 (7)	−0.0005 (5)	0.0059 (5)	−0.0007 (5)
N1	0.0155 (5)	0.0164 (5)	0.0120 (5)	0.0006 (4)	0.0037 (4)	0.0007 (4)
N2	0.0198 (6)	0.0265 (6)	0.0149 (5)	−0.0022 (4)	0.0034 (4)	0.0055 (4)
O1	0.0233 (5)	0.0275 (5)	0.0177 (5)	−0.0062 (4)	0.0052 (4)	0.0042 (3)
O2	0.0213 (5)	0.0314 (6)	0.0178 (5)	−0.0034 (4)	0.0009 (4)	−0.0008 (4)

### Geometric parameters (Å, º)

**Table:**

C1—C6	1.3942 (17)	C8—C9	1.3709 (16)
C1—C2	1.3949 (18)	C8—N1	1.3719 (15)
C1—C7	1.5159 (16)	C8—H8	0.9300
C2—C3	1.3886 (18)	C9—C10	1.4476 (16)
C2—H2	0.9300	C9—C12	1.4834 (16)
C3—C4	1.3933 (19)	C10—C11	1.3829 (16)
C3—H3	0.9300	C10—C13	1.4471 (17)
C4—C5	1.384 (2)	C11—N1	1.3516 (15)
C4—H4	0.9300	C11—H11	0.9300
C5—C6	1.3877 (18)	C12—O1	1.2375 (15)
C5—H5	0.9300	C12—N2	1.3374 (16)
C6—H6	0.9300	C13—O2	1.2253 (15)
C7—N1	1.4616 (14)	C13—H13	0.9300
C7—H7A	0.9700	N2—H2A	0.8600
C7—H7B	0.9700	N2—H2B	0.8600
			
C6—C1—C2	118.58 (11)	C9—C8—N1	109.08 (10)
C6—C1—C7	119.09 (11)	C9—C8—H8	125.5
C2—C1—C7	122.33 (11)	N1—C8—H8	125.5
C3—C2—C1	120.71 (12)	C8—C9—C10	106.34 (10)
C3—C2—H2	119.6	C8—C9—C12	121.46 (11)
C1—C2—H2	119.6	C10—C9—C12	132.19 (11)
C2—C3—C4	120.19 (12)	C11—C10—C13	119.54 (11)
C2—C3—H3	119.9	C11—C10—C9	106.24 (10)
C4—C3—H3	119.9	C13—C10—C9	134.08 (11)
C5—C4—C3	119.32 (12)	N1—C11—C10	109.15 (10)
C5—C4—H4	120.3	N1—C11—H11	125.4
C3—C4—H4	120.3	C10—C11—H11	125.4
C4—C5—C6	120.55 (12)	O1—C12—N2	122.68 (11)
C4—C5—H5	119.7	O1—C12—C9	120.65 (10)
C6—C5—H5	119.7	N2—C12—C9	116.67 (10)
C5—C6—C1	120.65 (12)	O2—C13—C10	127.90 (12)
C5—C6—H6	119.7	O2—C13—H13	116.0
C1—C6—H6	119.7	C10—C13—H13	116.0
N1—C7—C1	112.69 (9)	C11—N1—C8	109.20 (10)
N1—C7—H7A	109.1	C11—N1—C7	126.38 (10)
C1—C7—H7A	109.1	C8—N1—C7	124.07 (10)
N1—C7—H7B	109.1	C12—N2—H2A	120.0
C1—C7—H7B	109.1	C12—N2—H2B	120.0
H7A—C7—H7B	107.8	H2A—N2—H2B	120.0
			
C6—C1—C2—C3	−0.61 (18)	C12—C9—C10—C13	6.5 (2)
C7—C1—C2—C3	178.65 (11)	C13—C10—C11—N1	176.01 (10)
C1—C2—C3—C4	0.9 (2)	C9—C10—C11—N1	−0.30 (13)
C2—C3—C4—C5	−0.3 (2)	C8—C9—C12—O1	3.69 (18)
C3—C4—C5—C6	−0.6 (2)	C10—C9—C12—O1	−178.07 (11)
C4—C5—C6—C1	0.92 (19)	C8—C9—C12—N2	−176.06 (10)
C2—C1—C6—C5	−0.30 (18)	C10—C9—C12—N2	2.19 (19)
C7—C1—C6—C5	−179.59 (11)	C11—C10—C13—O2	−173.94 (12)
C6—C1—C7—N1	151.87 (11)	C9—C10—C13—O2	1.1 (2)
C2—C1—C7—N1	−27.39 (15)	C10—C11—N1—C8	0.01 (13)
N1—C8—C9—C10	−0.47 (13)	C10—C11—N1—C7	−173.30 (10)
N1—C8—C9—C12	178.17 (10)	C9—C8—N1—C11	0.30 (13)
C8—C9—C10—C11	0.47 (13)	C9—C8—N1—C7	173.80 (10)
C12—C9—C10—C11	−177.97 (12)	C1—C7—N1—C11	90.92 (13)
C8—C9—C10—C13	−175.05 (13)	C1—C7—N1—C8	−81.44 (13)

### Hydrogen-bond geometry (Å, º)

Cg1 is the centroid of the benzyl ring C1–C6.

**Table:**

*D*—H···*A*	*D*—H	H···*A*	*D*···*A*	*D*—H···*A*
N2—H2*B*···O2	0.86	1.99	2.8184 (14)	160
N2—H2*A*···O1^i^	0.86	2.22	3.0063 (14)	151
C8—H8···O1^ii^	0.93	2.69	3.4252 (15)	136
C7—H7*B*···O1^ii^	0.97	2.48	3.3123 (15)	144
C7—H7*A*···O2^iii^	0.97	2.66	3.3268 (15)	126
C11—H11···*Cg*1^iv^	0.93	2.58	3.4962 (14)	167

Symmetry codes: (i) −*x*+1, −*y*+2, −*z*−1; (ii) −*x*+2, −*y*+2, −*z*; (iii) *x*+1, *y*, *z*+1; (iv) *x*−1, *y*, *z*.
